# Barriers and Bridges: Black Doulas' Experiences in Healthcare Setting

**DOI:** 10.1111/birt.70034

**Published:** 2025-11-07

**Authors:** Sydnie Carraher, Kara Foster, Izziah Thabath, Ann Anderson‐Berry, Shannon Maloney

**Affiliations:** ^1^ University of Nebraska Medical Center, Department of Pediatrics, 981204 Nebraska Medical Center Omaha Nebraska USA; ^2^ University of Illinois at Chicago, School of Public Health, 1603 W. Taylor St. Chicago Illinois USA; ^3^ University of Nebraska Medical Center, Maurer College of Public Health, 984355 Nebraska Medical Center Omaha Nebraska USA

**Keywords:** black doulas, lived experience, quality improvement, racial disparities, systemic barriers

## Abstract

**Background:**

Black birthing people experience disproportionately high rates of adverse maternal and infant health outcomes. Doula support is associated with improved birth outcomes and can help reduce racial disparities, yet culturally congruent doulas face hurdles practicing in the healthcare setting. This quality improvement project aimed to understand the experiences of Black doulas in Nebraska to enhance integration into healthcare systems.

**Methods:**

Three group‐based interviews were conducted as part of a quality improvement project in August 2023 with six Black doulas practicing in Nebraska. Participants were recruited through community organizations and snowball sampling. Semi‐structured interviews explored doula practices, barriers, motivations, and sources of support. Transcripts were analyzed using thematic analysis.

**Results:**

Three main themes emerged: (1) Barriers inhibiting Black doulas in the healthcare setting, (2) Facilitators of a doula‐friendly clinical environment, and (3) Coping strategies. Doulas described facing resistance, stereotypes, and a lack of understanding about their role from some healthcare staff, contrasted with client advocacy. Self‐care strategies and peer support networks were critical for sustainable practice.

**Conclusion:**

Despite systemic barriers, Black doulas play a vital role in supporting Black families and addressing inequities. Recommendations include implementing doula‐friendly policies, addressing bias, fostering an inclusive environment in healthcare facilities, and enhancing the integration of culturally concordant doula support to improve outcomes for Black birthing people.

## Graphical abstract

Barriers and Bridges: Black Doulas’ Experiences in Healthcare Settings

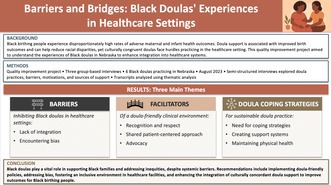

This quality improvement project explored the experiences of six Black doulas in Nebraska through semi‐structured interviews. Thematic analysis revealed three main themes: (1) Barriers including lack of recognition, integration, and bias; (2) Facilitators such as respect, shared patient‐centered approach, and advocacy; and (3) Coping strategies including support systems and maintaining physical health. Findings underscore the vital role Black doulas play in addressing maternal health inequities despite systemic barriers, with recommendations to implement doula‐friendly policies and enhance integration into perinatal healthcare systems.

## 1 Introduction

Adverse maternal and infant health outcomes disproportionately affect Black birthing people [1]. These individuals are at the highest risk for pregnancy‐related death, enduring rates two to three times higher than their White peers [1, 2]. Black birthing people also experience higher rates of labor interventions, cesarean birth, and preterm birth, leading to inequities in infant mortality as well [3, 4, 5]. Integration of doula support for Black birthing people is one strategy with the potential to improve maternity outcomes, thereby reducing health inequities [6].

Doulas are trained professionals who provide non‐medical physical, emotional, and informational support to clients and their families before, during, and after birth. Doula support is associated with improved birth outcomes and can have a positive impact on reducing racial disparities in maternal and infant health outcomes [7, 8]. The presence of a doula contributes to improved communication between clinicians, birthing people, and their families; improved quality of maternal care services; use of fewer obstetric interventions; less use of pain medications; reduced rates of perinatal mental health conditions; increased patient satisfaction; and the potential to reduce healthcare costs [6, 7, 9]. Culturally congruent doulas have the potential to reduce the impact of racism and bias by providing local, tailored, culturally appropriate, patient‐centered care and ensuring that the pregnant person has power and choice in their birthing journey [8].

Despite the benefits of doula support, barriers exist in accessing doula care, often due to a lack of knowledge about their role, cost, and availability of services. Among those who can access doula services, there are a limited number of culturally congruent doulas in the workforce [10]. Consequently, many Black birthing people at the highest risk for adverse outcomes are less likely to have access to and utilize doula services that could ameliorate some of that risk [9, 10].

Doulas also face challenges navigating and integrating into the healthcare system, including provider and nursing misconceptions of this role, inconsistent certification and training requirements, lack of service reimbursement, and resistance from healthcare team members [11, 12, 13, 14, 15]. The attitudes of obstetric providers and the power they hold within hospital settings present further challenges for doulas in providing care [16]. A lack of respect for the doula role and discord between doulas and clinical providers can lead to negative patient experiences and adverse clinical outcomes [11, 12].

In response to anecdotal concerns that Black pregnant people in Nebraska lack access to culturally congruent doula care, the state perinatal quality collaborative (PQC) [17] sought solutions to expand and diversify the doula workforce with the intention of reducing perinatal inequities. The state PQC partnered with I Be Black Girl (IBBG) [18], a community‐based reproductive justice organization, to launch a community‐engaged quality improvement (QI) initiative for high‐risk Black birthing people in September 2022. As part of this QI initiative, the PQC and IBBG conducted group‐based interviews with Black doulas to understand their lived experiences providing doula support in Nebraska and utilize this information to better prepare healthcare facilities for doula integration. This article reports on the qualitative data obtained from the doula group‐based interviews.

## 2 Methods

### 2.1 Project Design

As part of a QI initiative to integrate Black doulas into healthcare systems, we qualitatively analyzed group‐based interviews with Black doulas practicing in Nebraska. A community‐based participatory approach was used in the project's design and implementation. We engaged with community partner IBBG and Black doula collectives to develop the interview tool and format for the group‐based interviews.

Participants were recruited by IBBG, local community‐based Black doula organizations, and snowball sampling through email invitations and social media campaigns. Additionally, community partners promoted the interviews within their networks to solicit participants and increase community buy‐in. Doulas interested in participating were invited to complete an online form using a secure public link or QR code to determine eligibility. To be eligible, doulas needed to self‐identify as Black, be at least 19 years of age, practice as a doula in Nebraska, and have supported a client's birth within the past year. A representative from IBBG reviewed all form submissions to identify qualified candidates and contacted them to verify eligibility.

All group‐based interviews were conducted in August 2023. To ensure impartiality, the interviews were facilitated by a representative of IBBG, who was reflective of the Black community. Participants provided verbal informed consent before being interviewed and received a $100 gift card as compensation upon completion. This QI project was deemed exempt by the Institutional Review Board of a large midwestern public university.

### 2.2 Data Collection

Three group‐based interviews were conducted using Zoom (version 5.14), a video conferencing platform. With participants' verbal permission, each session was recorded and transcribed verbatim. As shown in Table 1, the interview guide addressed the following topics with participants: structure of personal doula practice, positive and negative experiences in Nebraska healthcare systems, motivations for becoming a doula, and sources of support to sustain their doula practice.

**TABLE 1:** Semi‐structured interview questions.

Operationalizing doula practice	Are you working alone or as part of a doula group/collective/part of an OB practice? How are you currently paid for your services (i.e., paid directly by client, barter/trade, and volunteer)? In your opinion, what is happening well in the landscape of doula practice in Nebraska? What is not going so well?
Experience	What types of challenges/barriers have you faced as a doula providing services in Nebraska? Please describe a time in which you had trouble getting into the patient's room. What was the nature of this barrier? How did you overcome it? Please describe a positive interaction you have had with other healthcare professionals while supporting a family as their doula. *Probe for specific location of experiences.
Motivation and support	Please tell us about your WHY. What supports do you have in place to sustain yourself and your doula practice?
Free to share	As you reflect on your experience as a doula interfacing with clinical settings, is there anything else you would like to share with us?

### 2.3 Data Analysis

A team of PQC staff conducted a thematic analysis of the interview transcripts, closely following the approach outlined by Braun and Clarke [19]. To begin the analysis process, team members independently created memos in Microsoft Word for each transcript, capturing initial impressions and thoughts. The team then collaboratively developed an initial codebook, including code families, subcodes, and code definitions, based on both inductive and deductive approaches. Transcripts and the initial codebook were transferred to Dedoose, a qualitative data analysis software, facilitating collaborative coding and theme identification. To ensure intercoder reliability, the team completed kappa tests within Dedoose, achieving excellent agreement (scores of 0.9 and 0.89). Throughout the coding process, regular team meetings were held to discuss coding discrepancies, ensure consensus in data interpretation, and refine emerging themes. The codebook underwent iterative revisions to capture emergent themes and refine code definitions. Upon completing the coding process, the team exported code summaries from Dedoose to synthesize the data and identify recurrent themes within each code family. Themes were refined through team discussion and consensus, ensuring that they accurately represented the data and captured the essence of the participants' experiences and perspectives.

## 3 Results

**FIGURE 1:**
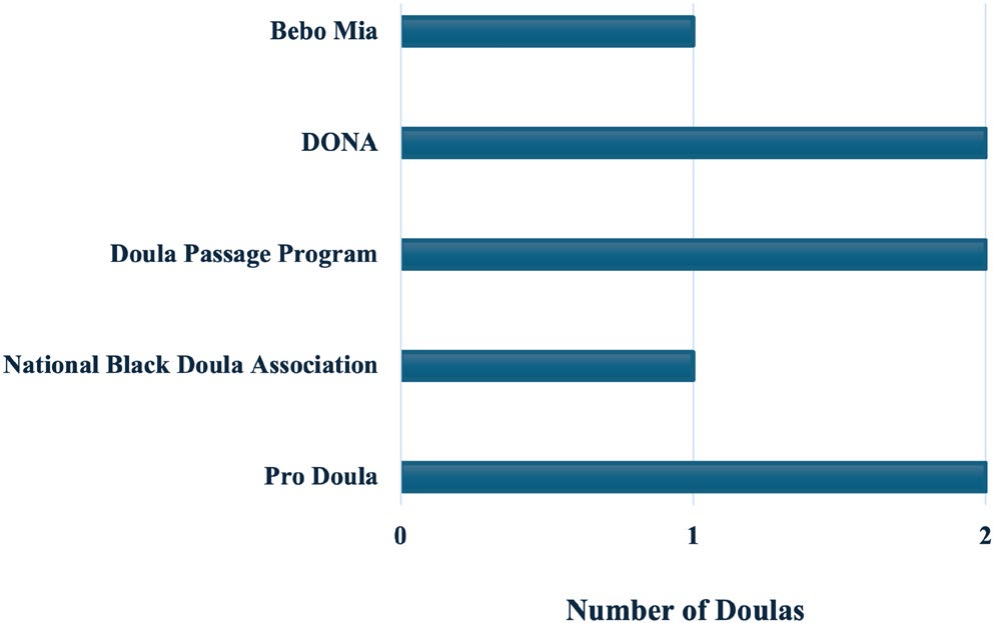
Doula training programs completed by participants. Some doulas participated in multiple training programs. [Colour figure can be viewed at wileyonlinelibrary.com]

Three group‐based interviews were conducted: two with multiple participants (*n* = 2, *n* = 3) and one individual interview. A total of six Black doulas participated in this project. Participants' ages ranged from 31 to 50 years, with varying levels of experience in doula practice and number of births attended in the past year. All participants had completed some formal doula training, as shown in Figure 1. Detailed demographic characteristics of the participants are presented in Table 2.

**TABLE 2:** Demographic characteristics of six participants.

Characteristics	*n*
Age in years	
31–40	3
41–50	3
Race ethnicity	
Black or African American	5
Black Hispanic	1
Years of doula practice	
1–3 years	5
4–6 years	1
Births participated in the past year	
1–5	1
6–10	2
11–15	2
20+	1

To describe the experiences of Black doulas in Nebraska, we categorized themes into three topics: (1) barriers inhibiting Black doulas in the healthcare setting, (2) facilitators of a doula‐friendly clinical environment, and (3) coping strategies.

### 3.1 Barriers Inhibiting Black Doulas in the Healthcare Setting

Black doulas described obstacles they encountered that hindered their work within healthcare settings. These challenges primarily manifested in two key areas: lack of integration and navigating bias.

#### 3.1.1 Lack of Integration

Despite the vital role that doulas play in helping their clients navigate the birthing experience, they were often not included as an important part of the health care team. Doulas described experiencing resistance from health care providers and staff. Multiple participants described instances where their clients advocated for doulas to be a part of their birthing experience despite being challenged by clinical staff:

One of the barriers that I've come across in some clinical settings is labor and delivery staff not valuing me as a key part of the support care team. … I've come across a nurse or two that [have] discounted my value in the room when the family that I'm supporting is like, “No, like you need to listen to her. She's advocating on our behalf.” Participant D

One of the most prominent challenges for doulas included barriers to room entry during labor and delivery, especially for cesarean births. Doulas described experiences in which they were asked to provide proof of certification at multiple hospitals, which served as an additional obstacle to a smooth birthing experience. One participant described an experience where they were denied entry to their client's cesarean birth and had to follow up with their client postpartum:

It just kind of depends on who the doctor is, but for the most part, they won't budge at all. They won't even let you wait in the patient's recovery room while they're back there getting the C‐section. …Rather [than] putting up a fuss and making a bad name for doulas, especially Black doulas, I just choose my battles. …It's not worth the fight, and it's not worth making doulas look bad because we're already fighting to be in this space. Participant A

Participants described challenges in how some medical professionals perceived the role of doulas. They reported instances where healthcare providers questioned the necessity of doulas, citing existing hospital staff as sufficient. This viewpoint, according to participants, overlooked the unique relationship between doulas and their clients. One doula elaborated on this perspective:

There's a struggle when you hear a doctor [say], “You don't need a doula, we got great hospital staff. The nurse can handle it.” It isn't about that. That nurse doesn't know that mother. That nurse doesn't know anything about that mom besides her medical history; she doesn't know what that mom likes, [what] comforts [her], and what makes her happy, [or] the struggles that she's been going through. And I think that's the problem that we have within our healthcare community. They're saying, “Oh well, doulas aren't necessary.” Yes, they are. They're so necessary to have for that mom; this mom is struggling; every birth is so different. Participant B

#### 3.1.2 Encountering Bias

Doulas reported experiencing various forms of bias in clinical settings, stemming from both professional reputation issues and racial prejudices. These biases create a complex landscape that doulas, particularly Black doulas, must navigate in their work. Participants consistently described encounters where their professional role was questioned or undermined, often in ways that seemed linked to their racial identity. One participant described this disparity:

I think it's mostly with BIPOC doulas that they're like that with … White women [doulas] are like, “I have a great experience.” But when it is a Black woman doula, they're like, “Dang, why are you here? Well, who are you supposed to be?” Participant B

Black doulas felt they were frequently mistaken for a friend or family member instead of an individual serving in a professional capacity. They found themselves in situations where their very presence in the clinical setting was questioned. One doula explained, “They're like, ‘Oh doulas, here they come,’ and then they see a Black doula. ‘Oh, okay, so who are you here for?’ They think you're just there. Like, no, I'm a doula” (Participant B).

Beyond race‐based biases, doulas also face challenges related to the overall reputation of their profession. One participant highlighted how the actions of some doulas can create barriers for the entire doula community:

I think another barrier is doula reputation. There might be people practicing as doulas who have tainted [our] reputation because of how they've entered spaces or communicated with providers and staff. So, a barrier sometimes is [the] reputation that precedes us, not because of my own doing, but because of someone else's. Participant D

### 3.2 Facilitators of a Doula‐Friendly Clinical Environment

Participants identified several facilitators that contributed to positive experiences in clinical settings, including recognition and respect, a shared patient‐centered approach, and advocacy from healthcare providers.

#### 3.2.1 Recognition and Respect

Doulas emphasized that positive experiences in clinical settings were strongly linked to being recognized and respected as valuable members of the healthcare team. Simple acts of acknowledgment, such as eye contact and courteous interactions, significantly impacted their sense of inclusion and worth. As one participant expressed, “Being acknowledged, being looked at in the eye, and shown respect. Not ignored or brushed off… [then] I felt welcome to be there and didn't feel like I was an imposition” (Participant C). These gestures of recognition not only improved the doulas' experience but also facilitated a more collaborative and effective care environment for their clients.

#### 3.2.2 Shared Patient‐Centered Approach

Doulas emphasized the importance of alignment between providers and doulas in delivering patient‐centered care. This synergy, rooted in non‐judgmental support and holistic care, enhanced both the birthing experience and the working environment. Doulas particularly valued providers who demonstrated attentiveness to patients' needs and concerns, offered non‐judgmental responses to patient inquiries, and practiced active listening while making space for patient voices. One participant highlighted the essence of this approach:

At the end of the day, it's not about me. It's about those moms. So, when [providers are] actually attentive to those moms' needs, answering [their] questions, and not getting irritated if the mom asks a question. …That's enough. Participant A

#### 3.2.3 Advocacy

Healthcare provider advocacy for doulas emerged as a crucial facilitator of a doula‐friendly environment. When faced with resistance, doulas valued having team members advocate for their presence. This advocacy involved providers actively intervening and affirming doulas' value and right to be in the delivery room. Such support enhanced doulas' ability to perform their role and reinforced their sense of belonging. One participant described an instance of strong advocacy:

I had to literally fight to get back. But then the midwife and the OB was like, “No, [Participant B]'s coming back here. We want her back here.” But it was the OR nurse that was giving me a dirty look the whole time, and the midwives saw it. She sent them to the OB. OB said, “…Before I make this incision, the doula is in here at my request and the request of this patient.” And she said, “If there's a problem, you are more than welcome to leave this OR.” That made me feel really good to know that there's someone there that was having my back. Participant B

While these facilitators improved doulas' experiences in clinical settings, participants also highlighted the importance of personal strategies for managing the inherent challenges of their role.

### 3.3 Coping Strategies

Several themes were identified that fell into the category of coping strategies.

#### 3.3.1 Need for Coping Strategies

Participants described the emotionally and physically demanding nature of doula work, reporting being on call at all hours and often spending extended periods providing intense physical and emotional support during labor and delivery. Participants identified various coping strategies to navigate the unique challenges they face as Black doulas. As well, doulas recognized that their own experiences of birth trauma could impact their work. Coping strategies were identified as an important part of preparing themselves to effectively support their clients, especially for doulas who had a history of traumatic birth.

When I went through training, it brought up things for me from my own birth experience, and understanding the role of a doula, understanding things that I didn't know in my own experience … Just making sure I was ready to step into that space so that I could be present for my client … Choosing my battles and thinking about what that looks like in my responses for all of us in this role. And so that self‐care is super important for me. Participant C

#### 3.3.2 Creating Support Systems

Doulas stressed the importance of maintaining personal boundaries and creating support systems to process the complex emotions that arise from their work:

Self‐care is so important because you don't realize you take on so much from that mom and that birth as a doula … And then [doulas are] going to carry all those emotions inside of them. So, I would advise them to speak to someone if you got to, because, at the end of the day, you're still human, and you're going to need to release that. Participant B

Doulas highlighted the value of mentorship programs and online doula communities in building supportive relationships crucial to sustaining their practice. Many also turned to professional mental health support, with some mentioning talk therapy as a valuable resource. Additionally, practices such as meditation and taking vacations were cited as important for maintaining emotional well‐being.

#### 3.3.3 Maintaining Physical Health

Recognizing the physical toll of their work, doulas emphasized the importance of caring for their bodies. They described a range of strategies to support their physical health, including staying hydrated, practicing yoga, seeking massage and chiropractic care, taking walks, consuming nutrient‐dense foods, and prioritizing rest. As one participant explained:

I tell people go for walks … That's something that can really help, just going for a walk. It doesn't matter where you're going. Just go out and get some fresh air, because having fresh air and breathing in this air it just helps to just relax from a birth. Participant B

## 4 Discussion

We sought to understand the experiences of Black doulas providing support to pregnant clients in Nebraska. Though evidence shows culturally congruent doulas can help reduce racial inequities and improve birth outcomes, doulas described healthcare settings that were not inclusive. For Black doulas, professional reputation challenges intersect with racial biases, potentially creating even more significant barriers to integration. They may find themselves needing to overcome both racial stereotypes and negative preconceptions about doulas in general, compounding the difficulties they face in clinical settings.

Black doulas often endure racist or biased care when advocating on behalf of their clients [20]. This shared identity and advocacy role can place an undue emotional burden on the Black doula [20]. Doulas anticipate and try to prevent scenarios that might cause undue emotional distress due to bias or racism and often work to prevent potentially contentious encounters. This unpaid emotional work goes beyond the scope of a doula's duties. The physiological impact of the clinical environment on Black doulas remains unquantified, warranting research as clinical spaces aim to be more welcoming and inclusive.

Clinical teams can facilitate the integration of doulas into the care team by displaying kindness and respect, administering patient‐centered care, and advocating for doulas' right to be present in birthing spaces. Doula work can be taxing by nature, but when doulas are welcomed into an inclusive birthing environment and allowed to practice within their full scope, their emotional and physical burdens may be minimized.

### 4.1 Recommendations

Culturally congruent doula support has been shown to improve birth outcomes for Black birthing people and babies [21]. To leverage this benefit, clinical teams should prioritize understanding the role of doulas and their positive impact on patient outcomes. Healthcare institutions should partner with doulas and patients to develop and publicly share doula‐friendly policies that protect against discrimination and ensure unrestricted access, such as allowing doulas into operating rooms for cesarean births without case‐by‐case judgment and eliminating certification proof requirements. These policies should be backed by systems to address staff or doula infractions promptly.

Beyond policy changes, clinical care teams should consciously address the culture of birthing environments. The medicalization of birth has allowed for the evolution of birthing environments that can feel sterile, impersonal, and unfriendly. Priority should be given to centering the patient and their birth experience, curbing power dynamics, and ensuring equitable interactions with doulas of varying backgrounds [22]. While providing equitable, respectful, and unbiased healthcare cannot eliminate systemic racism, clinical teams should strive to address personal biases and create safe, welcoming birth spaces for all. This can be achieved through ongoing education and training, including individual introspection exercises and group activities to develop shared expected practices [23]. Accountability measures such as regular assessments and corrective action plans can ensure adherence to anti‐racist and equitable practices. By integrating these recommendations, healthcare systems can cultivate inclusive environments that leverage doula support for improved maternal health outcomes and reduced inequities.

### 4.2 Strengths and Limitations

A key strength of this QI project lies in its community‐based participatory approach, which enhanced relevance, cultural sensitivity, and potential impact. By collaborating with community partners and Black doula collectives, we ensured our project aligned closely with community needs and perspectives. These ongoing partnerships facilitate the effective dissemination of findings back to the community and to healthcare systems, positioning us to guide QI initiatives and systems‐level changes.

A limitation of the project includes the small non‐probability convenience sample, which limits the generalizability of the findings. Participants identified as Black; consequently, their experiences may differ from doulas of other races, ethnicities, and geographic locations. Additionally, the sensitive subject matter and groupthink may have impacted the depth and breadth of participants' responses.

### 4.3 Implications

The Nebraska Perinatal Quality Improvement Collaborative is using these insights to support doula integration into healthcare settings. The barriers identified (lack of integration, bias, and professional reputation challenges) represent systemic issues likely affecting Black doulas across the country. Given evidence that doulas improve outcomes, especially among Black communities, addressing these barriers represents a critical opportunity to reduce maternal health disparities broadly. Healthcare facilities can address many obstacles through systematic changes, promoting awareness and acceptance of culturally congruent doula care. Implementing doula‐friendly policies, bias training, and inclusive birthing environments can improve patient satisfaction and outcomes while reducing healthcare inequities. Accountability measures should ensure adherence to these practices. Further efforts exploring doula‐provider dynamics in diverse communities and geographic locations will identify additional quality improvement opportunities.

## Conflicts of Interest

The authors declare no conflicts of interest.

## Data Availability

Research data are not shared.

## References

[1] D. L. Hoyert , “Maternal Mortality Rates in the United States, 2021,” NCHS Health E‐Stats, (2023), 10.15620/cdc:124678. 39946528

[2] S. L. Trost , J. Beauregard , F. Njie , J. Berry , A. Harvey , and D. Goodman , “Pregnancy‐Related Deaths: Data From Maternal Mortality Review Committees in 36 U.S. States, 2017–2019,” (2022), Centers for Disease Control and Prevention, U.S. Department of Health and Human Services.

[3] E. A. Howell , “Reducing Disparities in Severe Maternal Morbidity and Mortality,” Clinical Obstetrics and Gynecology 61, no. 2 (2018): 387–399, 10.1097/GRF.0000000000000349. 29346121 PMC5915910

[4] March of Dimes , “March of Dimes Report Card for Nebraska,” (2023).

[5] M. J. Osterman , B. E. Hamilton , J. A. Martin , A. K. Driscoll , and C. P. Valenzuela , “Births: Final Data for 2021,” National Vital Statistics Reports 72, no. 1 (2023): 122047, 10.15620/cdc:122047. 36723449

[6] C. Adams and S. P. Thomas , “Alternative Prenatal Care Interventions to Alleviate Black–White Maternal/Infant Health Disparities,” Sociology Compass 12, no. 1 (2017): e12549, 10.1111/soc4.12549.

[7] M. A. Bohren , G. J. Hofmeyr , C. Sakala , R. K. Fukuzawa , and A. Cuthbert , “Continuous Support for Women During Childbirth,” Cochrane Database of Systematic Reviews 7, no. 7 (2017): CD003766, 10.1002/14651858.CD003766.pub6. 28681500 PMC6483123

[8] J. A. Temple and N. Varshney , “Using Prevention Research to Reduce Racial Disparities in Health Through Innovative Funding Strategies: The Case of Doula Care,” Prevention Science 25, no. 1 (2024): 108–118, 10.1007/s11121-023-01497-2. 36757659 PMC11303420

[9] K. B. Kozhimannil , R. R. Hardeman , F. Alarid‐Escudero , C. A. Vogelsang , C. Blauer‐Peterson , and E. A. Howell , “Modeling the Cost‐Effectiveness of Doula Care Associated With Reductions in Preterm Birth and Cesarean Delivery,” Birth 43, no. 1 (2016): 20–27, 10.1111/birt.12218. 26762249 PMC5544530

[10] M. Sperlich , C. Gabriel , and N. M. St Vil , “Preference, Knowledge and Utilization of Midwives, Childbirth Education Classes and Doulas Among U.S. Black and White Women: Implications for Pregnancy and Childbirth Outcomes,” Social Work in Health Care 58, no. 10 (2019): 988–1001, 10.1080/00981389.2019.1686679. 31682786

[11] L. Lucas and E. Wright , “Attitudes of Physicians, Midwives, and Nurses About Doulas: A Scoping Review,” MCN: American Journal of Maternal Child Nursing 44, no. 1 (2019): 33–39, 10.1097/NMC.0000000000000488. 30531588

[12] K. Neel , R. Goldman , D. Marte , G. Bello , and M. B. Nothnagle , “Hospital‐Based Maternity Care Practitioners' Perceptions of Doulas,” Birth 46, no. 2 (2019): 355–361, 10.1111/birt.12420. 30734958

[13] New York City Department of Health , “The State of Doula Care in NYC 2023,” (2023), https://www.nyc.gov/assets/doh/downloads/pdf/csi/doula‐report‐2023.pdf.

[14] K. Knocke , A. Chappel , S. Sugar , N. De Lew , and B. D. Sommers , “Doula Care and Maternal Health: An Evidence Review. Office of the Assistant Secretary for Planning and Evaluation, U.S. Department of Health and Human Services,” December (2022), Issue Brief No. HP‐2022‐24, https://aspe.hhs.gov/sites/default/files/documents/dfcd768f1caf6fabf3d281f762e8d068/ASPE‐Doula‐Issue‐Brief‐12‐13‐22.pdf.

[15] Z. Sulaiman and M. Mullins , “Getting Doula's Paid: Advancing Community‐Based Doula Models in Medicaid Reimbursement Conversations,” Health Connect One; February 2023. Policy Brief.

[16] C. Adams and M. Curtin‐Bowen , “Countervailing Powers in the Labor Room: The Doula‐Doctor Relationship in the United States,” Social Science & Medicine 285 (2021): 114296, 10.1016/j.socscimed.2021.114296. 34365071

[17] “Nebraska Perinatal Quality Improvement Collaborative,” https://www.npqic.org.

[18] “I Be Black Girl,” https://www.ibeblackgirl.com.

[19] V. Braun and V. Clarke , “Using Thematic Analysis in Psychology,” Qualitative Research in Psychology 3, no. 2 (2006): 77–101, 10.1191/1478088706qp063oa.

[20] K. Thomas , S. Quist , S. Peprah , K. Riley , P. C. Mittal , and B. T. Nguyen , “The Experiences of Black Community‐Based Doulas as They Mitigate Systems of Racism: A Qualitative Study,” Journal of Midwifery & Women's Health 68, no. 4 (2023): 466–472, 10.1111/jmwh.13493. 37057730

[21] J. Mottl‐Santiago , D. Dukhovny , H. Cabral , et al., “Effectiveness of an Enhanced Community Doula Intervention in a Safety Net Setting: A Randomized Controlled Trial,” Health Equity 7, no. 1 (2023): 466–476, 10.1089/heq.2022.0200. 37731785 PMC10507922

[22] M. A. Stark , M. Remynse , and E. Zwelling , “Importance of the Birth Environment to Support Physiologic Birth,” Journal of Obstetric, Gynecologic, and Neonatal Nursing 45, no. 2 (2016): 285–294, 10.1016/j.jogn.2015.12.008. 26820356

[23] S. B. Garrett , L. Jones , A. Montague , et al., “Challenges and Opportunities for Clinician Implicit Bias Training: Insights From Perinatal Care Stakeholders,” Health Equity 7, no. 1 (2023): 506–519, 10.1089/heq.2023.0126. 37731787 PMC10507933

